# Psychopathy is a neglected public health problem

**DOI:** 10.3389/fpsyt.2025.1657080

**Published:** 2026-01-07

**Authors:** Melanie A. Chinchilla, Dennis E. Reidy, David S. Kosson

**Affiliations:** 1 Private Practice, San Francisco, CA, United States; 2 School of Public Health, Georgia State University, Center for Research on Interpersonal Violence, Georgia State University, Atlanta, GA, United States; 3 Department of Psychology, Rosalind Franklin University of Medicine and Science, North Chicago, IL, United States; 4 Department of Psychology, College of Health Professions, Rosalind Franklin University of Medicine and Science Chicago Medical School, North Chicago, IL, United States

**Keywords:** psychopathic, substance misuse, sexual risk, violence, public health

## Abstract

Although psychopathy has been widely studied within criminal justice contexts, its impact on society is rarely considered in public health contexts. Even so, evidence strongly implicates psychopathic traits in substantial costs (not just fiscal) to society. Individuals with psychopathic features commonly hurt those with whom they have relationships in important ways, including through abuse, manipulation, deception and, often, violence. We review the empirical evidence linking psychopathic traits to three recognized public health problems: substance misuse, sexual risk behavior, and violence. We argue that the evidence suggesting robust links between psychopathic traits and these known public health problems warrant the recognition of psychopathy as a public health concern in its own right. We argue that failing to consider psychopathy through a public health lens will slow research progress and the development of prevention strategies. Implications for public policy are considered.

## 1 Introduction

Although psychopathy has been widely studied within criminal justice contexts, its impact on society is rarely considered in public health contexts (1–3). Many individuals with psychopathic features seldom encounter the criminal justice system. Some appear able to operate successfully within the boundaries of conventional society for years in spite of behavior that adversely impacts the health of the people around them and of the social systems in which they live (4–6).

In contrast, violent acts, substance use, and sexual risk behavior are widely recognized public health threats that place individuals at heightened risk of injury, infection, or disease and that negatively impact the health of society. Psychopathic traits have often been linked to all three of these public health problems, and have been conceptualized as risk factors for these problems (e.g., 7, 8). Moreover, preliminary evidence suggests psychopathic traits may contribute to shared variance among public health threats (discussed below; see *Section 1.3*). We argue that the evidence for robust links between psychopathic traits and these public health threats suggests the possibility that psychopathy may account for some of the harms of these known public health concerns and is itself a significant threat to public health.

In addition, most contemporary approaches to preventing and treating these kinds of problems do not address psychopathy. However, recent research testing the effectiveness of intervention programs designed to impact youth with psychopathic traits provides preliminary evidence that these methods likely reduce the harm associated with psychopathic traits.

In short, we argue that psychopathic traits meet two important criteria for public health concerns: 1) they appear to have a substantial negative impact on the health of individuals and of society; and 2) preliminary evidence identifies promising avenues for effective prevention strategies. Consequently, increased effort is warranted to identify socio-behavioral risk and protective factors that are associated with the development of psychopathic traits, and to evaluate and implement prevention strategies that target these factors (9). Our intent is not to provide an exhaustive review of psychopathy and its relation to these particular outcomes, or health more broadly. Nor is it to critique these literatures, though we do attempt to rely on the most rigorous evidence available. Our purpose is to argue that psychopathy is a significant public health problem that warrants attention from public health and prevention researchers and practitioners and to illustrate the benefits to society likely to result from an increased public health response to psychopathy.

### 1.1 Psychopathy: definitions and prevalence

Psychopathy refers to a syndrome of personality pathology defined by a pattern of interpersonal, affective, and antisocial lifestyle characteristics including interpersonal manipulation, callous disregard for the rights of others, stimulation-seeking, poor anger control, impulsive, and persistent antisocial behaviors (10, 11). Psychopathic traits, like the features of most mental disorders exist on a continuum (12). People with high levels of these traits can be identified as people with psychopathy and have been identified in community samples in many different countries (e.g., 13).

Psychopathy is not the same thing as antisocial personality disorder (ASPD). Despite being described as synonymous with psychopathy in the Fifth Edition of the Diagnostic and Statistical Manual of Mental Disorders (DSM-5, 14), substantial research demonstrates these two syndromes are not equivalent (15, 16). Many individuals meet diagnostic criteria for ASPD but are not high in psychopathic traits, and a small minority of individuals with psychopathic traits do not meet diagnostic criteria for ASPD (17). Moreover, even among individuals with ASPD, those who also have psychopathic features exhibit more violent histories, poorer prognoses, and different kinds of emotion-processing and neurological anomalies than individuals with ASPD without psychopathic traits (15, 18–20). Herein, we focus our review and arguments on psychopathy. In several places, we distinguish findings pertaining to psychopathy from findings pertaining to ASPD (which is often comorbid with psychopathy). However, our arguments and conclusions pertain to preventing the public health harms associated with psychopathic traits because we believe scientific evidence suggests psychopathy is a public health problem.

Psychopathic traits can be assessed with a variety of different kinds of measures ranging from self-report measures (e.g., the Psychopathic Personality Inventory, the Triarchic Psychopathy Measure; 21, 22) to observational measures (23), to ratings by parents or teachers (24) and clinical measures (the Psychopathy Checklist measures; 25). Although the Psychopathy Checklist measures are widely considered the best validated measures of psychopathy (e.g., 26, 27; 28), all measures of psychopathy have limitations. In the sections that follow, we review studies that have employed a variety of different measures of psychopathy in a variety of different kinds of samples.

### 1.2 The public health impact of psychopathy

The adverse impact of psychopathy on public health is evident in its association with a myriad of risk behaviors and the widespread consequences of such behavior. To demonstrate this point, we review links between psychopathy and three classes of risk behaviors that receive attention in the field of public health: substance use, sexual risk behavior, and violence. We consider the social costs of these psychopathy correlates and their implications for considering psychopathy a public health concern. Although no direct evidence of causality has been identified for the vast majority of public health problems, psychopathic traits are commonly identified in childhood and adolescence, often prior to evidence of sexual behavior, substance use, or the age of criminal responsibility. The positive associations between psychopathic traits and these three kinds of problems have led researchers to posit psychopathic traits as risk factors for all three kinds of problems.

At the outset, we note that our chief focus is on the nature of bivariate relations between psychopathic traits and levels of substance use and misuse, sexual risk, and violent behaviors. The majority of the research we review reflects studies of male adults and adolescents (or samples that are primarily male) and includes studies with offender and community samples and a variety of different psychopathy measures. Most of the published studies were conducted with North American samples. Because psychopathy has been validated in youth, in women, and in populations from different countries and of different races and ethnicities, where possible, we note whether there is evidence that these relations generalize across age, biological sex, ethnicity/race, and/or country/culture.

#### 1.2.1 Substance use

Substance use is widely recognized as one of the costlier social problems facing the United States, with costs that include communicable and noncommunicable diseases, substance use disorders, other mental disorders, criminal activity, and injuries related to substance use (29–31). Substance use is also associated with reductions in work productivity, increased costs related to social services, treatment, and hospitalization, and increased stress and financial costs to family members (e.g., 32–34). Consequently, substance misuse harms not only individuals who misuse substances but also their families, the companies that employ these individuals, and the community institutions that allocate human and financial resources to treat substance misuse. The robust links between substance misuse and relapse (e.g., 35) increase the magnitude of these costs. Moreover, overdoses and deaths related to drug use have grown dramatically over the past ten years (36) although they may now be declining (37).

Links between psychopathy and substance misuse have been replicated across multiple independent samples (e.g., 38–40). Psychopathy is associated with a greater number of symptoms of substance dependence (41–43) and has been linked to a variety of adverse health outcomes in substance use disorder samples. Youth with psychopathic traits are prone to earlier onset of substance use and use of drugs from a larger number of substance classes (44, 45). Although most of the evidence is based on cross-sectional studies, longitudinal associations between psychopathic traits and substance misuse have also been reported (e.g., 44, 46, 47). Links between psychopathic traits and substance misuse generalize across gender, race or ethnicity, and nationality. Even so, some gender differences have been reported (see **Supplementary Material** for additional detail), and the correlations between psychopathy and substance misuse criteria range from small to large in magnitude.

In addition to individual harms associated with substance use, there is evidence that negative peer influences on substance use can extend to the friends of someone who misuses substances and even their social connections and other peers (48). In this context, individuals with psychopathic traits are reported to be more effective than others at persuading peers to engage in a variety of deviant behaviors including substance use (49–52).

According to a National Drug Intelligence Center study (NDIC), illicit substance use in the United States costs over $282 billion in 2023 dollars (53; updated from 2007 dollars). This estimate includes lost productivity and health costs. Individuals with ASPD reportedly comprise approximately 20% of those with alcohol and drug use disorders (54, 55). The conservative estimate that one third of those with ASPD are high in psychopathic traits (56) suggests psychopathic traits are likely to cost at least 18 billion dollars related to illicit substance use each year.^1^

#### 1.2.2 Sexual risk behavior

Sexual risk behavior has numerous consequences for the social, economic and physical well-being of individuals and populations (57). Participation in risky sexual behavior increases the likelihood of contracting several sexually transmitted infections (STIs) and diseases which themselves can lead to other life-threatening illnesses, including cancer and HIV (9). People who engage in sexual risk behaviors are also at heightened risk of transmitting STIs, especially those whose risk behaviors include sexual activity with a larger number of partners and sexual activity without using condoms. Sexually transmitted infections contribute in turn to long-term harm to the sexual and reproductive health of individuals and their partners and can predispose their children to lasting health complications which are associated with additional medical and hospitalization costs. One recent analysis estimates the total lifetime direct medical cost for new STIs in the U.S. in one year as approaching $20 billion annually (updated from 2018 dollars; 60).

Aside from these costs are the costs of unplanned pregnancies, especially among adolescents. Teen mothers are at heightened risk for complications including pre-term delivery, poor maternal weight gain, low infant birthweight, toxemia, neonatal mortality, and maternal mortality (61–63). In addition, girls with teen pregnancies have poorer educational and employment outcomes and lower socioeconomic status in adulthood (64–67). Moreover, the offspring of teen mothers face elevated risks for health complications (including low birth weight and infant mortality; 62) and heightened risk for some of the same adverse outcomes as their mothers, including adolescent pregnancies (67), lower. educational attainment, greater likelihood of unemployment, and poorer health as adults (61, 62, 64, 68). One analysis estimates the annual cost of teen pregnancy in the U.S. at approximately $13 billion dollars (updated from 2013 dollars; 69).

Substantial cross-sectional evidence links psychopathy to sexual risk behaviors in both men and women including hypersexuality, intercourse with a larger number of sexual partners, sexual affairs, and reduced use of condoms during sex (70–73). Among adolescents, psychopathic traits have been linked to earlier sexual debut, more unprotected sexual intercourse, and reduced willingness to modify risky behaviors (74–76). Psychopathic traits also predict future engagement in sexual risk behaviors (77; see also 78). Although these associations are quite variable in magnitude across sexual risk criteria and samples, similar links have been identified for studies conducted in North America and in other continents and for studies of psychopathy and gender (see *Supplementary Material* for additional detail). Psychopathy has also been linked to other unsafe behaviors, including sexual intercourse without condoms among HIV-positive males (79).^2^

Moreover, similar to theory and evidence reviewed above for the role of individuals with psychopathic traits in spreading substance use (e.g., 80), youth psychopathic traits appear to promote sexual risk behaviors among their peers, including early sexual debut, unprotected sexual behavior, and the use of sexual coercion, including engaging in sexual behavior with partners under the influence of alcohol or other substances that impair decision-making (71, 72, 78, 81–86). Psychopathic traits have also been linked with manipulative behaviors (including love bombing) designed to induce trust and promote romantic feelings, which may increase openness to sexual activity (e.g., 49, 88–90). All of these behaviors place individuals with psychopathic traits at heightened risk for both contracting and transmitting STIs to multiple peers, who may, in turn, transmit these infections to others (91–93).

Although it is difficult to estimate the costs of sexual risk behavior attributable to individuals with psychopathic traits, recent estimates suggest that the costs of STIs (e.g., lifetime costs of the STIs in one year estimated at $19 billion) and teen pregnancies in the United States ($13 billion annually) are substantial (see **Supplementary Materials** for details). A substantial proportion of youth without psychopathic traits also engage in sexual risk behavior, and estimates of the proportion of youth with psychopathic traits in the community vary widely (e.g., 94, 95; see **Supplementary Materials**). At the same time, studies of antisocial youth suggest one quarter to one half of these youth who report engaging in sexual risk behaviors appear to be characterized by at least moderate levels of psychopathic traits. Consequently, available estimates suggest the annual costs of sexual risk behavior by youth with high levels of psychopathy in the U.S. likely exceed $1 billion dollars. (This estimate does not include sexual risk behaviors committed by adults with psychopathic traits).

#### 1.2.3 Psychopathy and violence

Violence is a major contributor to premature death, disability, and injury and has long been recognized as a serious threat to public health (3, 96, 97). Over 150,000 people are hospitalized, and over 20,000 die each year as a result of violence in the U.S. alone (98–100). But the health consequences of violence also include increased risk for several psychological disorders among victims and survivors, including anxiety and depression, post-traumatic stress, and suicide (96, 101, 102). Those victimized by violence are also at heightened risk for poorer behavioral health, including participation in substance abuse and sexual risk behaviors that, in turn, place some individuals at heightened risk for STIs, unwanted pregnancies, overdoses, and death.

Moreover, violence victimization often exacerbates existing physical health problems, including cancer, cardiovascular disease, chronic pain, diabetes, gastrointestinal disorders, hypertension, pelvic inflammatory disease, and seizures (103). Further, youth who are victims or witnesses of violence are more likely to develop a range of interpersonal and social problems, including difficulties with emotion regulation, peer relationships, school adjustment, and pro-social behaviors (104–109). These kinds of physical and psychiatric problems are associated with increased costs to individuals and, through demands on healthcare, social services, and correctional institutions, increased costs to society. Such youth also more often endorse attitudes condoning violence (106, 110), increasing the likelihood of later acts of violence (106, 110).

Youth and adults with psychopathic traits are responsible for a disproportionate share of violence (1, 3, 6, 111, 112) and are therefore responsible for an outsized share of the adverse health and economic consequences. For example, Coid and Yang (1) reported that individuals with psychopathy comprised 0.7 percent of the general population of Great Britain but accounted for 17.5 percent of the violent incidents over a 5-year period. This finding suggests that developing effective violence prevention with fewer than 1 percent of the population could reduce collective levels of violence substantially. Psychopathic traits also correlate with severity of victim injury, number of different types of victims, and with perpetrating violence both inside and outside the home (1, 113–117). A study of over 500 U.S. community individuals aged 18 to 40 revealed a significant relation between psychopathic traits and perpetration of violence (11). Moreover, evidence suggests links between psychopathic traits and violent behavior generalize across gender, race or ethnicity, and nationality, although some gender differences and ethnicity differences have been reported, and these effects range from small to large in magnitude (see **Supplementary Material** for additional detail).

Although most studies are cross-sectional, psychopathic traits also predict the commission of violence prospectively. Within a large sample of civil psychiatric patients, Skeem and Mulvey (118) found psychopathy scores predicted future violence, with 50% of psychopathic patients committing a violent act during a one-year follow-up period compared to 22% of non-psychopathic patients. Similarly, among incarcerated individuals followed for several years, men with psychopathic traits were more likely than other men to be charged with new violent crimes (119–122).

There are few comprehensive estimates of the costs of violent crime in the U.S. However, two recent estimates place the annual costs in the hundreds of billions. Anderson (123, 124) updated his own earlier estimates for the annual costs of rape, robbery, and assault to $346.6 billion (updated from 2020 dollars) and provided estimates for murders that permit a rough estimate of the annual cost of murders in the U.S. at $480.3 billion (see **Supplementary Materials** for details). Others have estimated that violence-related health care, lost work days, and reduced productivity cost the global economy billions of U.S. dollars per year (96, 125). One recent estimate for the annual costs of medical treatment and lost productivity due to homicide and nonfatal assaults was approximately $437 billion in the U.S. (100).

Taken together, these estimates suggest violent crime costs Americans upwards of $800 billion per year. The proportion of violent crime due to people with psychopathy can be estimated using the same formula previously used by Kiehl and Hoffman (126) to estimate the proportion of crime due to psychopathy. They argued that 20% of the crime reflected people with psychopathy. Given the substantial evidence that individuals with psychopathic traits account for a disproportionate share of the violent crime, it appears conservative to rely on the same figure in estimating the proportion of violent crime attributable to psychopathy. If individuals high in psychopathy account for only 20% of the costs of violent crime, then the costs of violence associated with psychopathy in the U.S. appear to exceed $150 billion annually (see **Supplementary Materials** for additional detail).

This figure likely underestimates the costs of violence by individuals with psychopathy. Even studies with methods designed to estimate the costs for those who do not report crimes (e.g., 123) do not commonly capture the harms to the intimate partners, children, and other family members and friends of those with psychopathic traits who witness the abuse of others (127).

#### 1.2.4 Additional costs to the health of society

There is also evidence that the negative impact of psychopathy extends to include harm to the overall health of societies in additional ways. We note here two examples.

First, beyond the harm to individuals through violence at their workplaces, people with psychopathic traits harm workplaces and organizations themselves (128). It is difficult to quantify the financial and reputational harm done to larger systems such as companies (through workplace-related lawsuits, corporate theft, and fraud) or to public institutions (through corruption and abuse of government programs). However, there is evidence psychopathic traits predict not only abusive individual interactions with individual supervisees, but also reduced job satisfaction among coworkers (129, 130), reductions in productive work behavior (131–133), and damage to corporate climates (130, 134).

Second, in addition to the harm to those in relationships with people with psychopathy, it appears likely (but not yet certain) that there is harm to children who depend on those relationships. First, the links between psychopathic traits and sexual infidelity and a larger number of live-in and marital relationships can help to explain the associations between psychopathic traits and reduced marital satisfaction and divorce (135). The links between psychopathy and emotional, physical, and sexual abuse appear likely to also contribute to unstable marriages, but abusive behavior is also expected to have a large negative impact on partners’ health. Damage to a child’s parent (i.e., a parent without psychopathy) is harmful to that child because traumatic experiences reduce people’s effectiveness (e.g., 136, 137). It has been argued that parents with psychopathic features also negatively impact their children’s development (138, 139). Although there are few direct studies, in one sample, psychopathic traits in fathers were associated with poor parenting and with greater behavior problems in children (140). In addition, one longitudinal study demonstrated psychopathic traits in fathers were associated with psychopathic traits in their offspring (141) although these could reflect genetic mechanisms (142). Such links suggest there are likely to be additional negative public health impacts for those exposed to parental figures with psychopathic traits during childhood. However, such repercussions are difficult to quantify.

It is sometimes argued that evidence for genetic factors makes environmental factors (including interventions) unimportant. The evidence that genetic factors contribute to the development of psychopathic traits does not rule out the utility of intervention efforts in reducing the levels of psychopathic traits that emerge over time. As in other areas of psychopathology, most behavior genetic studies of psychopathy find consistent evidence of nonshared environmental effects but not of shared environmental effects (142, 143). In addition, there is evidence that environmental factors can interact with genetic factors. For example, some research has found that parenting practices by an adoptive mother buffered against the heritable risk for callous-unemotional (CU) features (144, 145). These findings demonstrate there is substantial room for environmental impacts in genetic models.

### 1.3 Pleiotropic effects of psychopathy on public health

Although substance misuse, sexual risk behavior, and violence represent distinct public health problems, there is evidence suggesting they may be related. Notably, increased sexual risk behaviors and increased violence have been observed in individuals with substance misuse (e.g., 146–148). Similarly, violence and substance use have been identified as challenges faced by individuals with sexual risk behaviors (149–151), and substance misuse and sexual risk have been identified as challenges among individuals who commit violence (e.g., 152–154). Moreover, although most intervention programs are designed to address a single problematic behavior, some intervention programs have been designed to impact some combination of these public health problems (e.g., see 155–158). These relations suggest the possibility that some external factor could be related to all three of these public health problems.

In recent years, a few studies have reported important relations among psychopathic traits, sexual risk behavior, and substance use. Thornton and colleagues (77) reported that substance use appears to partly account for the relations between psychopathic traits in adolescence and risky sexual behavior. There is also preliminary evidence that psychopathy in adults contributes to the shared variance between violence and substance misuse. Examining a large sample of incarcerated male detainees, Vincent et al. (159) reported that psychopathic traits accounted for a significant proportion of the variance shared between indices of violent behavior and dependence symptoms for several classes of substances. These findings suggest the possibility that psychopathic traits may also confer heightened risk for several other kinds of risky or harmful activities.^3^ In this context, it is plausible that people with psychopathic traits may contribute further to the harms to which their offspring, siblings, parents, friends, romantic partners, colleagues, and organizations are exposed. Because most studies examine costs related to only one outcome at a time, the true cost burden of psychopathy is likely greater than even current estimates suggest. Studies are needed to provide more comprehensive estimates of the cost burden of psychopathy.

## 2 Psychopathy and interventions for addressing these public health problems

Many intervention programs have been designed to combat substance misuse, sexual risk behaviors, and violence. Recognition of these as public health problems has contributed to substantial investments in preventing and treating these kinds of harmful public health behaviors. In this section, we focus on two specific issues.

First, it appears that most interventions designed to ameliorate substance misuse not explicitly include procedures for addressing people with psychopathic traits. Reviews of treatments for substance use disorders that we identified did not mention psychopathy explicitly (e.g., 161–166). Several reviews have suggested comorbid psychopathology increases the difficulty of treating substance misuse (167, 168), and several have emphasized poorer outcomes for those with a comorbid personality disorder (156, 169, 170).

Similarly, most interventions designed to promote sexual health or reduce sexual risk behaviors do not address psychopathy (e.g., 149, 171–174). We were unable to identify reviews of sexual risk behavior that addressed personality disorders in general. However, some reviews and some individual studies have identified links between specific personality disorders or related traits (e.g., impulsivity) and sexual risk behavior (e.g., 175–177).

Many reviews of violence interventions do not address psychopathy or personality disorders as relevant factors (e.g., 178–184). Reviews that include personality disorders have emphasized that comorbid personality disorders are associated with lower rates of program success, including reduced motivation and greater dropout (e.g., 185–187). However, only a small proportion appear to include studies that address psychopathy explicitly (e.g., 188–190).

There is also little attention to people with psychopathic traits in the empirical literatures focusing on substance misuse and sexual risk behaviors. Notably, most review papers focused on impacting substance misuse and sexual risk behaviors do not explicitly address psychopathy (e.g., 191–198). (In the area of violence, some reviews do explicitly address psychopathy, e.g., 199). Even so, because people with psychopathic traits are more likely than others to misuse alcohol and other psychoactive substances, to engage in sexual risk behaviors, and to inflict violence on others, interventions that reduce psychopathy appear to represent an efficient way to reduce multiple public health problems. From this perspective, interventions that reduce the prevalence of psychopathic traits (or reduce the costs associated with psychopathic traits) may also reduce the costs associated with these recognized public health problems.

Second, the few studies that have evaluated the impact of psychopathic traits on the effectiveness of interventions for some of these public health problems have reported that these traits are associated with reduced effectiveness of programs designed to reduce unhealthy behaviors, including violence (200, 201; see also 202). Among forensic samples, the negative impacts of psychopathic traits on treatment compliance and working alliance appear more robust than the negative impact of ASPD on treatment outcomes (e.g., 203; cf., 204). Studies often (but not always) indicate the core affective and interpersonal components of psychopathy (those which differentiate psychopathy from ASPD; 205) are most predictive of treatment non-compliance and smaller gains in treatment (59, 201, 206, 207; cf. 200).^4^ Findings that psychopathic traits moderate the effectiveness of interventions designed to mitigate these costs indicate that such interventions are more effective for individuals without these traits than for those with them.

## 3 Decreasing the burden associated with psychopathy

Although psychopathy has sometimes been considered untreatable (56, 208, 209), recent research has provided evidence of the value of two different kinds of methods for reducing the harms associated with psychopathy. First, methods are available that appear to aid in reducing the development of psychopathic traits. Second, methods are available for reducing the harms caused by people who have developed psychopathic traits. A range of possible prevention strategies for reducing these impacts of psychopathic traits are summarized in **Table 1**.

**Table 1:** Prevention Strategies.

**Primary Prevention Strategies** ▪ Developing behavioral conditioning interventions for young children to positively reinforce prosocial behaviors ▪ Emotion training for children ▪ Parent training to increase parental warmth for and reduce early parental rejection/abuse of children with emotional and behavioral difficulties ▪ Educating preschool teachers to cope more effectively with youth displaying callous and aggressive behaviors ▪ Improving parent – teacher coordination for youth exhibiting incipient behavior problems ▪ Implementing social norms interventions to prevent corporal punishment **Secondary Interventions** ▪ Parenting intervention for youth who are displaying psychopathic traits: Parent-Child Interaction Therapy adapted for CU traits (210, 211) **Tertiary Interventions** ▪ Treatment strategies based on the intervention developed by the Mendota Mental Health Institute: using behavioral contingencies (with additional treatment factors) to reinforce prosocial behavior in youth with psychopathic traits (e.g., 212, 2011) ▪ Treatment strategy developed by Ribeiro da Silva et al. (213, 214), based on compassion- focused therapy, for youth with psychopathic traits ▪ Treatment strategies based on the intervention developed by Olver et al. (206), which employs cognitive behavioral approaches to addressing criminogenic needs in high risk- high needs offenders, including adults with psychopathic traits ▪ Treatment strategies based on the High-Risk Intervention Programme of Wilson and colleagues (215, 216) designed to produce behavior and personality change in incarcerated adults with psychopathy through a transparent framework and focus on the impact of behavior patterns on outcomes, including support for participants’ prosocial behaviors, building motivation through discussion of realistic aspirations, dealing with negative and sabotaging behaviors, and encouraging self-observation and reflection. The program also emphasizes support for program staff. ▪ Treatment strategies based on the Chromis Violence Reduction Program originally designed to help offenders deemed dangerous and personality disordered in His Majesty’s Prison Frankland (United Kingdom) to identify unhelpful schemas and learn cognitive skills to facilitate their development of prosocial goals (217).

### 3.1 Factors that help to reduce the severity of psychopathic traits

Studies suggest that factors which promote the security of parent-child attachment and which promote constructive parenting can help to reduce the development of psychopathic traits over time. As early as 2003, Frick et al. reported that families characterized by lower levels of “negative parenting” demonstrated greater declines in psychopathic traits over a four-year follow-up period. More recent studies indicate individual differences in parenting quality are prospectively predictive of changes in the levels of psychopathic traits children display. Many of these studies have focused only on the affective component of psychopathy (often referring to the affective traits as callous unemotional or CU traits)(218); even so, they demonstrate that greater parental involvement and positive reinforcement, and less parental harshness are predictive of lower levels of specific psychopathic traits in children years later 219).

### 3.2 Opportunities for prevention

Recent studies also provide preliminary evidence for effective intervention in clinical samples of youth with psychopathic traits. Several researchers had earlier reported evidence that youth with conduct problems and CU traits are responsive to nurturance and rewards but are less responsive to punishments than other youth with conduct problems (208, 220). Consistent with this perspective, Caldwell et al. (212) reported that incarcerated youth offenders with psychopathic features showed significant gains after periods of intensive treatment in a setting where contingencies were designed to reward prosocial behavior. In this program, a behavioral point system ensured participants were rewarded each day for good behavior displayed the previous day. Youth high in psychopathic traits who spent a greater proportion of their time at this treatment center were less likely to violently reoffend than those who resided primarily in conventional youth prisons (212). In addition, Caldwell and colleagues (222), provided evidence that such treatments may reduce levels of psychopathic traits (at least as measured by adolescents’ self-reports). Even so, apart from replications with independent samples of youth at the same institution, it does not appear this approach has been examined in an independent sample. Of course, these interventions reflect a response to psychopathy after it has manifested and engendered problems, a tertiary prevention approach. However, identifying the effective elements in these interventions may yield fruitful starting points for adapting them to primary and secondary prevention strategies.

There is some evidence that early interventions may help to inhibit the development of psychopathic traits. McDonald and colleagues (223) demonstrated that a program designed to improve the parent-child relationship, increase prosocial behavior, and reduce problem behaviors led to reductions in parent-reported psychopathic traits compared with a control condition in which parents could seek out support or referrals, and these differences were maintained during a follow-up period. More recently, Kimonis et al. (211) reported that participation in an enhanced form of parent-child interaction therapy (Parent-Child Interaction Therapy adapted for CU traits) led to reductions in the affective features of psychopathy among preschoolers (mean age 4.5); this finding has been replicated in independent samples (210, 224). Collectively, this evidence suggests that some kinds of early interventions may help to prevent the development of some of the problematic behaviors (e.g., violence, substance use, sexual risk behavior) that are associated with high levels of psychopathic traits (see also 225). These findings also suggest that further investments in developing interventions targeting parent behavior early in childhood are likely to be helpful for reducing the costly and destructive behavior associated with psychopathy. Further, given the evidence that some early environmental risk factors are reliably associated with increases in the likelihood of psychopathic traits, interventions that reduce these risk factors may help to prevent the development of psychopathic traits. At the same time, it remains important to examine the robustness of these findings in independent samples and with investigators other than those who developed the intervention programs.

These examples are provided only to demonstrate the gains that have been made towards effective intervention to reduce the development of psychopathic traits and the costs associated with psychopathic traits. They are not intended to serve as *the* proposed solutions to psychopathy and its harmful effects. Our goal is not to lay out what a public health prevention approach to psychopathy would look like in specificity here. Rather, we are proposing that shifting our perspective from a primarily criminal justice orientation to an integrated public health perspective will offer important opportunities, methods, and resources to abate this societal issue.

## 4 Limitations

Because the focus of this review was direct relations between psychopathic traits and well-recognized public health concerns, we did not investigate the possibility that other important variables moderate the strength or direction of the relations that emerged from our review. Although the links between psychopathic traits and substance misuse, sexual risk behaviors, and violent behaviors have been replicated across different kinds of measures of psychopathy and in different kinds of samples (e.g., forensic vs. community settings, samples in different countries, samples that differ in gender or ethnicity or race; e.g., 70, 73, 226–228; see **Supplementary Materials**), there is also is evidence that some of the relations between psychopathy and these harmful behaviors are impacted by other factors (e.g., 229–231). It remains possible that unexamined factors may moderate the strength of these relations. Further, every measure of psychopathy has limitations.^5^ In addition, most measures of psychopathic traits include items that tap into behaviors associated with some of the public health problems reviewed here (e.g., stimulation seeking associated with use of psychoactive substances; promiscuity including sexual relationships with multiple partners, and reactive or proactive violence). Most studies we reviewed did not control for predictor-criterion overlap in assessing the relations reported (112); consequently, the magnitude of the relations may be smaller than they appear. Moreover, some of the direct effects we discussed were relatively small in size (e.g., 229, 232, 233). Nevertheless, the aggregate burden of psychopathy on population health is substantial and necessitates applying a public health approach to prevent it.

## 5 Summary and conclusions

Psychopathy places a substantial burden on societal institutions. Psychopathic traits adversely impact not only individuals with high levels of psychopathic traits themselves but also their families, friends, co-workers, victims, and the organizations in which they function. Less severe, on average, but still substantial are the impacts of individuals with moderate levels of psychopathic traits in the same relationship contexts (e.g., 234). Psychopathy presents a unique challenge for mental health practitioners, medical providers, and law enforcement personnel. Psychopathic traits are associated not only with high levels of risky behaviors, but with reductions in the effectiveness of some treatments (e.g., 59, 201, 206).

The current analysis suggests the public health burden of psychopathy is substantial. However, preliminary evidence suggests the potential to lessen or prevent this burden: 1) by reducing levels of psychopathic traits among children at risk for developing psychopathy, and 2) by reducing the costs of violence among adolescents and adults with psychopathic traits. Adaptation and effectiveness tests of programs based in part on these interventions as primary prevention and additional secondary prevention strategies may be the next step to increasing the use of methods that can reduce the development of psychopathic traits. Our intention is not to advocate for a particular intervention or set of interventions. Our goal is to promote the perception of psychopathy under a public health lens in order to stimulate broadly research and the development of effective prevention strategies.

Psychopathic traits have been linked with individual harm in a variety of different kinds of personal relationships and professional relationships (e.g., 5, 129, 235 Preston et al. (236). In the absence of effective policies for managing deception, organizations are at risk for disruption and victimization. To protect their stockholders, organizations may benefit from developing policies to be able to manage both staff members and clients who practice deception and manipulation. Yet few organizations or institutions have developed policies to address situations in which individuals misrepresent their goals and their behavior.

As research continues to demonstrate the value of identifying those with psychopathic traits, public health experts and stakeholders may at some point wish to consider the merits of screening for psychopathic traits in specific contexts. Ethical considerations regarding when and how to engage in such screening practices are critical. It is important that any assessments of psychopathic traits in youth are conducted for the benefit of those youth and their families and that screening is not used to deny opportunities to youth. For example, it may be possible to direct additional resources towards some youth (e.g., child management strategies emphasizing positive reinforcement rather than punishment) without using labels that link the resources to psychopathy (237). In addition, resources are needed to educate people about how to protect themselves from people with such features in these contexts. Education about psychopathy should help to reduce the costs of psychopathy. In sum, greater recognition of the harmful impact of psychopathy on society as a whole and application of a public health approach will accelerate the development and dissemination of prevention strategies and policies that help systems and individuals function more effectively.

In writing this manuscript, we have presented a selection of evidence to demonstrate why psychopathy should be a public health priority. It was not our intention to be exhaustive in reviewing these literatures. For example, beyond the impacts on individuals with psychopathic traits and those who interact with them in close relationships, psychopathy may additionally impact public health through financial (e.g., Ponzi Schemes), corporate (ENRON), or political malfeasance (acts contributing to genocide; 4, 134). Further, the mental and physical health of employees may be adversely affected by supervisors with psychopathic traits who create hostile work environments (131). However, the true nature and burden of psychopathy will remain unknown as long as we continue to address the issue as though it is solely a criminal justice problem. We propose that psychopathy is a public health problem influencing the health and well-being of individuals across the social ecology.

## Data Availability

The raw data corroborating statements made in the text of this article can be found at https://www.osf.io/8rdbu and https://www.osf.io/845n9.

## Footnotes

1 These estimates do not include all substance misuse-related costs. Notably, they do not include costs stemming from the misuse of alcohol; substantial evidence links psychopathic traits to more severe symptoms of alcohol dependence (e.g., 41, 43, 47). In addition, some psychopathic traits are associated with less successful completion of treatment for substance misuse (58, 59) which is likely to increase costs.

2 Some of these correlates are reported to be different in cisgender male than in cisgender female individuals (87).

3 In contrast, a similar study reported that alcohol misuse was not associated with the link between ASPD and intimate partner violence (160).

4 Preliminary evidence suggests that psychopathic traits also reduce the effectiveness of interventions designed to deter corruption, another threat to public health (221).

5 One of the chief limitations of self-report measures of psychopathy is that self-report measures of the affective and interpersonal features of psychopathy correlate only very modestly with clinical indices of these traits (e.g., 26). The PCL measures tend to be more difficult to use, requiring substantial training and time and reviews of collateral material (often institutional files).
