# Supplementary material for: Psychopathy is a neglected public health problem
Source: Front Psychiatry. 2026 Jan 7;16:1657080. doi: 10.3389/fpsyt.2025.1657080 (PMC12819179; doi:10.3389/fpsyt.2025.1657080)
Supplement: Supplementary file 1 [file Supplementaryfile1.pdf]

Supplementary Materials

In this document, we provide additional detail on some issues raised in the manuscript. First, we provide greater detail about the bases for the statements in the paper that attempt to provide or apply cost estimates to selected public health problems associated with elevated levels of psychopathic traits. Second, we provide additional detail about the generality of the relations between psychopathic traits and substance misuse, sexual risk behavior, and violent behavior across factors such as development, gender, ethnicity and race, and national affiliation.

1. Estimating Selected Costs Associated with Psychopathic Traits

The information provided here is not intended to provide a formal economic analysis but to demonstrate how we derived the rough estimates for the economic burden of psychopathy in the domains of substance misuse, risky sexual behavior, and violent crime reported. To facilitate comprehension, we have converted all cost estimates provided in prior studies to December, 2023 dollars using the Consumer Price Index (CPI) Calculator (1).

Estimating the Contribution of Psychopathic Traits to the Costs of Substance Misuse

The estimate of the annual cost of substance misuse was based on a report from the National Drug Intelligence Center (2). Their estimate that substance misuse cost the United States $193 billion in 2007 dollars translates to an estimated $281.9 billion in 2023 dollars. Assuming that individuals with antisocial personality disorder (ASPD) account for one fifth of those costs (e.g., 3) and that individuals high in psychopathic traits comprise approximately one third of those with ASPD (4) results in an estimate of $18.8 billion.

Estimating the Contribution of Psychopathic Traits to Sexual Risk Behavior in Youth

Only a crude estimate of the costs of sexual risk behavior attributable to individuals with

psychopathic traits can be calculated based on available data. The estimated lifetime costs of the STIs that occur in one year in the United States have been estimated conservatively at $19.0 billion (in 2023 dollars, updated from 2019 dollars; 5). The estimated annual cost of the teen pregnancies that occur in one year have been estimated at $13.2 billion (updated from 2010 dollars; 6). Moreover, these estimates do not include the lifetime costs of teen pregnancies or the costs of sexual risk behavior resulting in adult pregnancies.

Most STIs and teen pregnancies appear to be consequences of risky sexual behavior. However, in light of a report suggesting that adolescents may intentionally become pregnant in approximately 23.5% of cases (7-8), we selected 76.5% of the estimated cost burden to approximate the costs of STIs and teen pregnancies due to sexual risk behavior. Sexual assaults also account for a small proportion of teenage pregnancies (5.0%; 7) and are included as reflecting sexual risk behavior by perpetrators.

Examination of available samples of youth with psychopathic traits indicates a high percentage of individuals who engage in risky behavior are also moderately high (or higher) in psychopathic traits. Notably, among a large sample of justice-involved youth from three diverse US communities ($N = 1178$), individuals endorsing callous-unemotional traits comprised 47.3% of those who reported engaging in unprotected sexual intercourse (9; see also 10). In a smaller sample of 107 adolescent detainees, 27.1% of those who reported engaging in sexual risk behavior ($n = 48$) received Psychopathy Checklist: Youth Version (PCL:YV; 11) ratings of 30 or higher indicating substantial levels of psychopathic traits; 41.7% were characterized by moderate levels of psychopathic traits (PCL:YV total scores ≥ 25; 12). These findings suggest a substantial proportion of the youth engaging in risky sexual behavior are moderate or higher in

psychopathic traits. However, community estimates suggest that risky sexual behavior among adolescents is relatively common, with some studies reporting one sixth of 14-year-olds and up to one third of 15-year-olds have initiated sexual intercourse (e.g., 13-16). At the same time, the wide range of estimates of the prevalence of youth with callous-unemotional traits in community samples makes it difficult to estimate the contribution of psychopathy with precision. Prevalence estimates range from about 9% to 27% for self-report, parent- and teacher-report measures (depending on cutoffs used; 17) but are below 1% for studies using PCL measures (11).

Nevertheless, the associations between psychopathic traits and risky sexual behavior suggest that the small minority of youth with psychopathic traits engages in a disproportionate share of the risky sexual behavior that occurs among young adolescents. This disproportionate share of the burden is even greater if one takes into account the increased likelihood of sexually assaultive behavior by youth with psychopathic traits which also increases the risk of sexually transmitted illnesses and unwanted pregnancies.

These studies demonstrate the importance of additional studies of the contribution of youth with psychopathic traits to sexual risk behavior in community samples, ideally using multiple measures of psychopathic traits. Even so, a range of estimates for the annual costs of psychopathic traits to sexual risk behavior in the US can be identified. Using estimates based on the costs of sexually transmitted illnesses alone, the annual costs attributable to individuals with high levels of psychopathic traits appear likely to exceed $1 billion per year, and, if youth with moderate levels of psychopathic traits account for a substantial proportion of sexual risk behavior, the annual costs may be as great as $6 billion.

$$0.765 \times \$19.0 \text{ billion} \times .09 = \$1.3 \text{ billion}$$

0.765 X $19.0 billion X .27 = $3.9 billion

0.765 X $19.0 billion X .42 = $6.1 billion

As noted above, these estimates exclude the contributions of people with psychopathic traits to adult pregnancies resulting from sexual risk behavior.

Estimating the Contribution of Psychopathic Traits to Violent Crime

There are few comprehensive estimates of the annual costs of violent crime in the US. Anderson (18) reported updated estimates for the annual costs of rape, robbery, and assault of $346.6 (updated from 2020 dollars; 19). Estimates he provided for murders (based on 20-21) yield an estimated annual cost of murders in the US of $480.3 billion (in 2023 dollars). Summing across these kinds of violent crime yields an annual estimate of $826.9 billion (in 2023 dollars).

More concretely, the above figure of $480.3 billion for the cost of murders was based on two estimates of the cost per murder cited by Anderson (18) multiplied by the estimated number of murders in the US during one year. McCollister et al. (21) had estimated the cost per murder based on jury awards after subtracting economic costs to victims at $12.5 million per murder (updated from 2020 dollars). In contrast, attempting to take into account costs to victims, to offenders, and to society, DeLisi et al. (20) had estimated the cost at $24.4 million / murder (updated from 2020 dollars). A simple average of these estimates yields an estimate of the cost per murder at $18.5 million. The number of murders in the US per year was estimated as 26,031 information provided by the National Center on Health Statistics (22), yielding an estimated annual cost of murders in the US of $480.3 billion.

In estimating the extent to which people with psychopathic traits contribute to the costs of overall crime, Kiehl and Hoffman (23) had relied on the common finding that adults with

psychopathy (as measured by the Psychopathy Checklist-Revised) constitute at least 20% of most prison and jail populations (e.g., 24). As summarized in text (see *Introduction*), there is substantial evidence that individuals with psychopathic traits account for a disproportionate share of violent crime. Consequently, it appears conservative to rely on the same 20% estimate (that Kiehl and Hoffman used) to estimate the proportion of *violent* crime attributable to psychopathy. If individuals high in psychopathy account for only 20% of the costs of violent crime in the US, the likely annual cost appears to reach $165 billion.

$826.9 billion X .20 = $165.4 billion

2. Considering the Generality and Robustness of the Relations Between Psychopathic Traits and Selected Public Health Problems

Prior to comparing psychopathy findings for different samples that vary in ethnicity/ race or gender or other important factors, it is important to examine whether the same syndrome exists in different kinds of samples. For example, researchers often try to demonstrate that a construct (such as a syndrome, i.e., psychopathy) is invariant in different ethnic groups, and they distinguish different levels of invariance. The establishment of configural invariance and metric invariance are often considered sufficient. Configural invariance means scores on items covary or cluster together in roughly the same dimensions in two different groups so that associations between different features of psychopathy appear to fit the same patterns. Metric invariance means the items have roughly the same loadings on the superordinate construct; in short, in different groups (e.g., European Americans and African Americans), the items are contributing

to the same degree to the systematic variance associated with psychopathy. There can be even greater degrees of equivalence; in some cases, each of the items is also at its most discriminating at the same levels of the overall syndrome (scalar invariance), and even the item residuals are sometimes similar (residual invariance).

Several studies report invariance in the psychopathy construct from adolescence through adulthood (e.g., 25-27). Several studies have reported invariance in the psychopathy syndrome as assessed with clinical measures across race or ethnicity (28-30). Although some studies have reported metric invariance in male and female samples, they have also suggested some difference in the levels of psychopathic traits at which some items are most discriminating (31-32) or in the nature of psychopathy for men versus women (e.g., 33). Nevertheless, most researchers assume there is sufficient invariance to permit the kinds of comparisons summarized here. Of course, evidence for invariance does not ensure that correlations with external variables will be similar, only that it makes sense to compare them. In this context, we summarize the robustness of correlations between measures of psychopathic traits and indices of substance misuse, sexual risk behavior, and violent behavior.

Substance Misuse

The associations between psychopathy and substance misuse have been reported to be relatively similar in male and female samples (e.g., 34; see also 35). In spite of the generally robust positive relations between overall psychopathic traits and substance misuse outcomes, there is limited evidence that Factor 1 traits or the affective component of Factor 1 may be protective against severity of substance misuse symptoms (e.g., 36-37) and that this effect may be more general among women than among men (38). The links between psychopathic traits and substance misuse (or a related construct) have also been demonstrated in samples from a variety of different countries, including studies in South America (39), Africa (40), Europe (41-43), and Asia (44). Similarly,

some studies have reported largely similar relations in samples for participants in different ethnic or racial groups (e.g., 45-46).

Sexual Risk Behavior

We have focused to some extent on sexual risk behavior among adolescents in this review. There are robust links between psychopathic traits and a similar range of sexual risk behaviors among adults, including greater number of partners, increased likelihood of extramarital affairs, unsafe sexual activity (including lower use of contraception), and higher rates of perpetrating sexual aggression (47-53; cf. 54).

Similarly, psychopathic traits are associated with an elevated prevalence of promiscuous behavior and deviant sexual interests in male and female adolescents and adults (55-56) although some relations have been reported to be stronger in male or in female participants (55-56). In at least one study, correlations between sexual assault behaviors and psychopathy were generally stronger among women than among men (57). In another study, only some of the links with sexual risk behaviors appeared robust to statistical controls (55). Links between psychopathic traits and sexual risk behaviors have been reported for study participants in Asian (58) and European (56) countries.

Violence

Links between psychopathic traits and violent behavior have been reported in studies of youth (e.g., 59-61) and adults (e.g., 62-63), in correctional and community samples (e.g., 64). Consistent with gender differences in violent behavior, some studies report stronger links to relational aggression in female than male participants with psychopathic traits (65). Even so, studies have reported that psychopathic traits are robustly associated with violent behavior in both males and females (e.g., 60, 65-69). Although most studies of psychopathy and violence have employed participants from North America, studies of samples from other continents have replicated similar relations (e.g., 66, 69-74), as

have studies assessing psychopathy in European American versus African American offenders (e.g., 75-76). Some studies suggest that links between psychopathic traits and violence are weaker in Latin American offenders than in European American and African American offenders (e.g., 76-77).

References Cited

1. Consumer Price Index Inflation Calculator. United States Bureau of Labor Statistics. Available at: https://www.bls.gov/data/inflation_calculator.htm (Accessed December 19, 2023).

2. National Drug Intelligence Center. The economic impact of illicit drug use on American society. Washington, DC: United States Department of Justice; 2011. Available at: https://www.justice.gov/archive/ndic/pubs44/44731/44731p.pdf (Accessed November 10, 2016).

3. Goldstein RB, Compton WM, Pulay AJ, Ruan WJ, Pickering RP, Stinson FS, et al. Antisocial behavioral syndromes and DSM-IV drug use disorders in the United States: Results from the National Epidemiologic Survey on Alcohol and Related Conditions. Drug Alcohol Depend. (2007) 90:145–58. https://doi.org/10.1016/j.drugalcdep.2007.02.023

4. Hare RD, Neumann CS. Psychopathy: Assessment and forensic implications. Can J Psychiatry. (2009) 54:791–802. https://www.10.1177/070674370905401202

5. Chesson HW, Spicknall IH, Bingham A, Brisson M, Eppink ST, Farnham PG, et al. The estimated direct lifetime medical costs of sexually transmitted infections acquired in the United States in 2018. Sexually Transmitted Dis. (2021) 48:215–21. https://www.10.1097/OLQ.0000000000001380

6. Power to Decide (formerly The National Campaign to Prevent Teen and Unplanned Pregnancy). Counting it up: Key data (2013). Washington, DC. Available at: https://powertodecide.org/what-we-do/information/resource-library/counting-it-key-data

7. Holmes MM, Resnick HS, Kilpatrick DG, Best CL. Rape-related pregnancy: Estimates and descriptive characteristics from a national sample of women. Am J Obstetrics Gynecology. (1996)

175:320–5. https://www.ajog.org/article/S0002-9378(96)70141-2/abstract

8. Rosengard C, Phipps MG, Adler NE, Ellen JM. Adolescent pregnancy intentions and pregnancy outcomes: A longitudinal examination. J Adolesc Health. (2004) 35:453–61. https://www.sciencedirect.com/science/article/pii/S1054139X040005169.

9. Frick PJ. Unpublished analyses of callous-unemotional traits and risky sexual behavior among participants in the CrossRoads Study. (2023).

10. Thornton LC, Frick PJ, Ray JV, Wall Myers TD, Steinberg L, Cauffman E. Risky sex, drugs, sensation seeking, and callous unemotional traits in justice-involved male adolescents. J Clin Child Adolesc Psychol. (2019) 48:68–79. https://www.10.1080/15374416.2017.1399398

11. Forth AE, Kosson DS, Hare RD. The Hare Psychopathy Checklist: Youth Version, technical manual. New York: Multi-Health Systems, Inc (2003).

12. Kosson, D. (2025, May 28). Unpublished adolescent data collected for study of psychopathic traits and risky sexual behavior. Retrieved from osf.io/845n9

13. Centers for Disease Control and Prevention. Youth risk behavior surveillance —United states. Morbidity Mortality Weekly Rep. (2010) 59. Available at: https://www.cdc.gov/mmwr/pdf/ss/ss5905.pdf.

14. Cheedalla A, Moreau C, Burke AE. Sex education and contraceptive use of adolescent and young adult females in the United States: An analysis of the National Survey of Family Growth 2011–2017. Contraception: X. (2020) 2:100048. https://pmc.ncbi.nlm.nih.gov/articles/PMC7732995

15. Madkour AS, Farhat T, Halpern CT, Godeau E, Gabhainn SN. Early adolescent sexual initiation as a problem behavior: A comparative study of five nations. J Adolesc Health. (2010) 47:389–98. https://pmc.ncbi.nlm.nih.gov/articles/PMC2945604

16. Zhang X, Liu Q, Wang X, Vasilenko SA. Unpacking the impact of early adverse childhood

experiences on early onset of sexual intercourse among an urban birth cohort of early adolescents. J Adolesc Health. (2023) 73:1110–6. https://pmc.ncbi.nlm.nih.gov/articles/PMC10842965/

17. Kemp EC, Frick PJ, Matlasz TM, Clark JE, Robertson EL, Ray JV, et al. Developing cutoff scores for the Inventory of Callous-Unemotional Traits (ICU) in justice-involved and community samples. J Clin Child Adolesc Psychol. (2023) 52:519–32. https://pubmed.ncbi.nlm.nih.gov/34424103/

18. Anderson DA. The aggregate cost of crime in the United States. J Law Econ. (2021) 64:857–85. https://chicagounbound.uchicago.edu/jle/vol64/iss4/7

19. Cohen MA, Miller TR, Rossman SB. The costs and consequences of violent behavior in the United States. In: Reiss AJ Jr., Roth JA, editors. Understanding and preventing violence, Vol. 4. Consequences and control. National Academy Press, Washington, DC (1994).

20. DeLisi M, Kosloski A, Sween M, Hachmeister E, Moore M, Drury A. Murder by numbers: Monetary costs imposed by a sample of homicide offenders. J Forensic Psychiatry Psychol. (2010) 21:501–13. https://www.researchgate.net/publication/238317331_Murder_by_numbers_Monetary_costs_imposed_by_a_sample_of_homicide_offenders?

21. McCollister KE, French MT, Fang H. The cost of crime to society: New crime- specific estimates for policy and program evaluation. Drug Alcohol Depend. (2010) 108:98–109. https://pmc.ncbi.nlm.nih.gov/articles/PMC2835847/

22. Curtin SC. Trends in death rates for leading methods of injury: United States, 2003–2023. NCHS Data Brief. (2025) Mar;526. doi:10.15620/cdc/174582

23. Kiehl KA, Hoffman MB. The criminal psychopath: History, neuroscience, treatment, and economics. Jurimetrics. (2011) 51:355.

24. Hare RD. The Hare Psychopathy Checklist-Revised: Technical Manual. North Tonawanda, NY: Multi-Health Systems (2003).

25. Hawes SW, Byrd AL, Kelley SE, Gonzalez R, Edens JF, Pardini DA. Psychopathic features across development: Assessing longitudinal invariance among Caucasian and African American youths. J Res Pers. (2018) 73:180-188. https://doi.org/10.1016/j.jrp.2018.02.003

26. Neumann C, Wampler M, Taylor J, Blonigen DM, Iacono WG. Stability and invariance of psychopathic traits from late adolescence to young adulthood. J Res Pers. (2011) 45(2):145-152. https://doi.org/10.1016/j.jrp.2010.12.003

27. Ngo DA, Neumann CS, Maurer JM, Harenski C, Kiehl KA. Psychopathic traits in adult versus adolescent males: Measurement invariance across the PCL-R and PCL:YV. Behav Sci (Basel). (2024) 14(8):672. https://doi.org/10.3390/bs14080672

28. Botha R, Polaschek DLL, Wilson N. Measurement invariance and convergent validity of the Psychopathy Checklist: Screening Version (PCL:SV) across New Zealand Māori and European samples. J Pers Assess. (2025). https://doi.org/10.1080/00223891.2025.2460992

29. Cooke DJ, Kosson DS, Michie C. Psychopathy and ethnicity: Structural, item, and test generalizability of the Psychopathy Checklist—Revised (PCL-R) in Caucasian and African American participants. Psychol Assess. (2001) 13:531–42. https://doi.org/10.1037/1040-3590.13.4.531

30. Horan JM, Brown JL, Jones SM, Aber JL. Assessing invariance across sex and race/ethnicity in measures of youth psychopathic characteristics. Psychol Assess. (2015) 27:657–68. https://doi.org/10.1037/pas0000043

31. Spormann SS, Mokros A, Schneider S. Structural differences in psychopathy between women and men: A latent modeling perspective. Forensische Psychiatrie Psychologie Kriminologie. (2023) 17:174–88. https://doi.org/10.1007/s11757-023-00765-9

32. Tsang S, Schmidt KM, Vincent GM, Salekin RT, Moretti MM, Odgers CL. Assessing psychopathy among justice involved adolescents with the PCL:YV: An item response theory examination across gender. Pers Disorders: Theory Research Treat. (2015) 6:22–31. https://doi.org/10.1037/per0000094

33. Strand S, Belfrage H. Gender differences in psychopathy in a Swedish offender sample. Behav Sci Law. (2005) 23:837–50. https://doi.org/10.1002/bsl.674

34. Sellbom M, Donnelly KM, Rock RC, Phillips TR, Ben-Porath YS. Examining gender as moderating the association between psychopathy and substance abuse. Psychology Crime Law. (2017) 23:376–90. https://doi.org/10.1080/1068316X.2016.1258466

35. Maurer JM, Edwards BG, Harenski CL, Kiehl KA. Psychopathic traits are associated with lifetime history of nicotine dependence among incarcerated offenders. Subst Use Misuse. (2023) 58:444–53. https://doi.org/10.1080/10826084.2023.2167495

36. Brieman CL, McGarrigle WJ, Cope LM, Kiehl KA, Kosson DS. Clarifying relationships between core features of psychopathy and substance (mis)use: A replication and extension in two large independent samples. J Pers Disord. (2024) 38:138–56. https://doi.org/10.1521/pedi.2024.38.2.138

37. Cope LM, Vincent GM, Jobelius JL, Nyalakanti PK, Calhoun VD, Kiehl KA. Psychopathic traits modulate brain responses to drug cues in incarcerated offenders. Front Hum Neurosci. 2014;8:87. https://doi.org/10.3389/fnhum.2014.00087

38. Schulz N, Murphy B, Verona E. Gender differences in psychopathy links to drug use. Law Hum Behav. (2016) 40:159–68. https://doi.org/10.1037/lhb0000165

39. Silva AR, Relva IC, Simões M. Impacto da vinculação e dos traços de psicopatia nos comportamentos aditivos dos adolescentes [Impact of attachment and traits of psychopathy on addictive behaviors of adolescents]. Rev Interamericana Psicología. (2020) 54:1–29.

https://doi.org/10.30849/ripijp.v54i1.1161

40. Yitayih Y, Soboka M, Tesfaye E, Abera M, Mamaru A, Adorjan K. A cross-sectional study of psychopathy and khat abuse among prisoners in the correctional institution in Jimma, Ethiopia. PloS One. (2020) 15(1):e0227405. https://doi.org/10.1371/journal.pone.0227405

41. Charalampous K, Stavrinides P, Georgiou S, Giasemidou P. Is there a psychopathic element in adolescent drinking? A longitudinal study. Eur J Dev Psychol. (2019) 16:47–65. https://doi.org/10.1080/17405629.2017.1334547

42. Coid J, Yang M, Ullrich S, Roberts A, Hare RD. Prevalence and correlates of psychopathic traits in the household population of Great Britain. Int J Law Psychiatry. (2009) 32:65–73. https://doi.org/10.1016/j.ijlp.2009.01.001

43. Gustavson C, Ståhlberg O, Sjödin A-K, Forsman A, Nilsson T, Anckarsäter H. Age at onset of substance abuse: A crucial covariate of psychopathic traits and aggression in adult offenders. Psychiatry Res. (2007) 153:195–8. https://doi.org/10.1016/j.psychres.2006.12.020

44. Shariat SV, Assadi SM, Noroozian M, Pakravannejad M, Yahyazadeh O, Aghayan S, et al. Psychopathy in Iran: A cross-cultural study. J Pers Disord. (2010) 24:676–91. https://doi.org/10.1521/pedi.2010.24.5.676

45. Kosson DS, Smith SS, Newman JP. Evaluating the construct validity of psychopathy in Black and White male inmates: Three preliminary studies. J Abnorm Psychol. (1990) 99(3):250-259. https://doi.org/10.1037/0021-843X.99.3.250

46. Sullivan EA, Abramowitz CS, Lopez ML, Kosson DS. Reliability and construct validity of the Psychopathy Checklist-Revised for Latino, European American, and African-American male inmates. Psychol Assess (2006) 18:382-392.

47. Benotsch EG, Rodríguez VM, Hood K, Lance SP, Green M, Martin AM, et al. Misleading

sexual partners about HIV status among persons living with HIV/AIDS. J Community Health. (2012) 37:1049–57. https://doi.org/10.1007/s10900-011-9529-5

48. Campos MD. Psychopathy, alcohol use, and AIDS related sexual behavior. Washington State University (2003). [dissertation]. Pullman, WA: Washington State University; 2003.

49. Knight RA, Guay JP. The role of psychopathy in sexual coercion against women: An update and expansion. In: Patrick CJ, editor. Handbook of psychopathy. Guilford Press, New York (2018). p. 662–81.

50. Kosson DS, Kelly JC, White JW. Psychopathy-related traits predict self-reported sexual aggression among college men. J Interpersonal Violence. (1997) 12:241–54. https://journals.sagepub.com/doi/10.1177/088626097012002006

51. Reynolds BL, Maurer JM, Cook AM, Harenski C, Kiehl KA. The relationship between psychopathic traits and risky sexual behavior in incarcerated adult male offenders. Pers Individ Dif. (2020) 156:109798. https://doi.org/10.1016/j.paid.2019.109798

52. Tourian K, Alterman A, Metzger D, Rutherford M, Cacciola JS, McKay JR. Validity of three measures of antisociality in predicting HIV risk behaviors in methadone-maintenance patients. Drug Alcohol Depend. (1997) 47:99–107. https://doi.org/10.1016/S0376-8716(97)00076-8

53. Zara G, Bergstrøm H, Farrington DP. The sexual life of men with psychopathic traits. J Criminological Research Policy Pract. (2021) 7 :164–78. https://doi.org/10.1108/JCRPP-04-2020-0036

54. Timmermans E, De CaluwéE, Alexopoulos C. Why are you cheating on tinder? Exploring users' motives and (dark) personality traits. Comput Hum Behav. (2018) 89:129–39. https://doi.org/10.1016/j.chb.2018.07.040

55. Cook AM, Maurer JM, Reynolds BL, Harenski CL, Kiehl KA. Psychopathy and risky sexual behavior in incarcerated women. Criminal justice Behav. (2022) 49:1456–73.

https://journals.sagepub.com/doi/10.1177/00938548221105057

56. van Bommel R, Uzieblo K, Bogaerts S, Garofalo C. Psychopathic traits and deviant sexual interests: The moderating role of gender. Int J Forensic Ment Health. (2018) 17:256–71. https://doi.org/10.1080/14999013.2018.1499684

57. Hoffmann AM, Verona E. Psychopathic traits and sexual coercion against relationship partners in men and women. J Interpersonal Violence. (2021) 36:NP1788– 809. https://doi.org/10.1177/0886260518754873

58. Chan HO, Beech A. Risky sexual behavior and psychopathy: Testing the relationship in a non-clinical sample of young adults in Hong Kong. Behav Sci (Basel Switzerland). (2024) 14:94. https://doi.org/10.3390/bs14020094

59. Asscher JJ, van Vugt ES, Stams GJ, Deković M, Eichelsheim VI, Yousfi S. The relationship between juvenile psychopathic traits, delinquency and (violent) recidivism: A meta-analysis. J Child Psychol psychiatry Allied disciplines. (2011) 52:1134–43. https://doi.org/10.1111/j.1469-7610.2011.02412.x

60. Bauer DL, Whitman LA, Kosson DS. Reliability and construct validity of Psychopathy Checklist: Youth Version scores among incarcerated adolescent girls. Crim Justice Behav. (2011) 38(10):965-987.https://doi.org/10.1177/0093854811418048

61. Brazil KJ, Forth AE. Adolescent psychopathic traits, early adversity, and intimate partner violence. Criminal Justice Behav. (2024) 51:213–29. https://doi.org/10.1177/00938548231219810

62. Holper L, Ehrbar C, Fancellu F, Hauser NC, Habermeyer E, Mokros A. Criterion Validity of the Psychopathy Checklist in Legal Contexts: An Updated Meta-Analysis. J Pers Assess. (2025) 107(5):547-562. https://www.researchgate.net/publication/389560708_Criterion_Validity_of_the_Psychopathy_Checkli

st_in_Legal_Contexts_An_Updated_Meta-Analysis

63. Moretti G, Flutti E, Colanino M, Ferlito D, Amoresano L, Travaini G. Recidivism risk in male adult sex offenders with psychopathic traits assessed by PCL-R: A systematic review. Medicine Science Law. (2024) 64:41–51. https://journals.sagepub.com/doi/10.1177/00258024231187186

64. Reidy DE, Zeichner A, Seibert LA. Unprovoked aggression: Effects of psychopathic traits and sadism. J Pers. (2011) 79:75–100. https://pubmed.ncbi.nlm.nih.gov/21223265/

65. Colins OF, Fanti KA, Salekin RT, Andershed H. Psychopathic personality in the general population: differences and similarities across gender. J Pers Disord. (2017) 31:49–74. https://biblio.ugent.be/publication/8588082

66. Lee V, Egan V. Predictors of aggression in Southeast Asian female prisoners. Pers Individ Dif. (2013) 54:113−117. https://doi.org/10.1016/j.paid.2012.08.024

67. Orue I, Calvete E, Gamez-Guadix M. Gender moderates the association between psychopathic traits and aggressive behavior in adolescents. Pers Individ Dif. (2016) 94:266–71. https://doi.org/10.1016/j.paid.2016.01.043

68. Mager KL, Bresin K, Verona E. Gender, psychopathy factors, and intimate partner violence. Pers Disord. (2014) 5:257–67. https://pmc.ncbi.nlm.nih.gov/articles/PMC4175448

69. Weizmann-Henelius G. Våldsbenägna kvinnor i Finland - Personlighet och livssituation [Violent female offenders in Finland - Personality and life situation. Psykologia. (2005) 40:177–80.

70. Maneiro L, Cutrín O, Gómez-Fraguela XA. Gender differences in the personality correlates of reactive and proactive aggression in a Spanish sample of young adults. J Interpersonal Violence. (2022) 37:NP4082–107. https://pubmed.ncbi.nlm.nih.gov/32924770/

71. Onyedibe MCC, Ifeagwazi CM, Ugwu DI. Psychopathy and aggressive behaviour among Nigerian male prison inmates: The moderating role of substance abuse. J Psychol Afr. (2018) 28:365–

70. https://doi.org/10.1080/14330237.2018.1523342

72. Santos-Hermoso J. Psychopathic femicide: The influence of psychopathy on intimate partner homicide. J Forensic Sci. (2022) 67:2061–6. https://pmc.ncbi.nlm.nih.gov/articles/PMC9314048/

73. Schmitt R, Pinto TP, Gomes KM, Quevedo J, Stein A. Psychopathy personality in a sample of young Brazilian offenders. Arch Clin Psychiatry. (2006) 33:297–303. https://doi.org/10.1590/S0101-60832006000600002

74. Wang M.−C., Gong J, Gao Y, Zhang X, Yang W, Luo J. Variants of psychopathy in Chinese male offenders: A latent profile analysis in a large prison sample. J Criminal Justice. (2020) 69:Article 101708. https://doi.org/10.1016/j.jcrimjus.2020.101708

75. Fanti KA, Lordos A, Sullivan EA, Kosson DS. Cultural and ethnic variations in psychopathy. In: Patrick CJ, editor. Handbook of psychopathy, 2nd ed. The Guilford Press (2018). p. 529–569).

76. Walsh Z. Psychopathy and criminal violence: The moderating effect of ethnicity. Law Hum Behav. (2013) 37:303–11. https://pubmed.ncbi.nlm.nih.gov/23181487/

77. Spraberry Tekell CD. Multicultural study of psychopathy: An examination of Latin American differences. ScholarWorks@UTEP, University of Texas at El Paso (2012).
